# Supplementary material for: HAPLN1 potentiates peritoneal metastasis in pancreatic cancer
Source: Nat Commun. 2023 Apr 24;14:2353. doi: 10.1038/s41467-023-38064-w (PMC10126109; doi:10.1038/s41467-023-38064-w)
Supplement: Supplementary file 1 — Supplementary Information [file 41467_2023_38064_MOESM1_ESM.pdf]

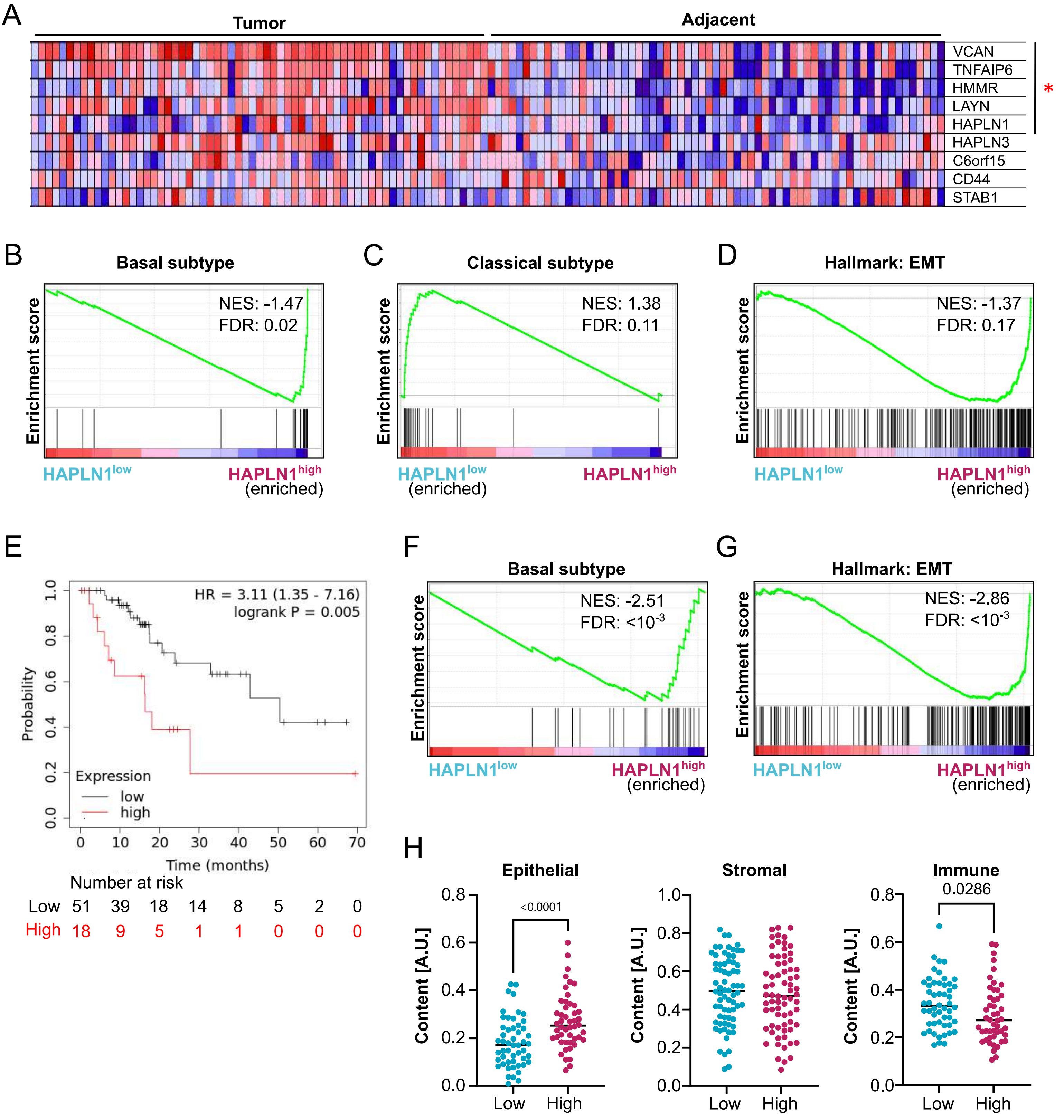

Supplementary Figure 1

Supplementary Figure 1: HAPLN1 is upregulated in PDAC tissue and associated with worse disease outcome

A. GSEA on GSE62452 (n=61 normal adjacent and n=69 PDAC tumor samples, (23)) on "Gene ontology for Hyaluronic Acid binding". Leading edge genes significantly deregulated are marked with red star. **B-D.** GSEA on GSE50827. Before analysis PDAC patients were divided in *HAPLN1* high/low according to mean *HAPLN1* expression. Gene sets of "Basal subtype" (**B**), "Classical subtype" (**C**) or "Hallmark of Epithelial-to-Mesenchymal transition (EMT)" (**D**). n=69. **E**. Regression free survival plot using the KM-plotter tool and analyzing TCGA dataset. **F.** GSEA on TCGA dataset. Before analysis PDAC patients were divided in *HAPLN1* high/low according to mean *HAPLN1* expression. Gene sets of "Basal subtype" (**F**) or "Hallmark of Epithelial-to-Mesenchymal transition" (**G**). **H**. Cell component content analysis from the dataset published by Cao et al. [23]. Stromal: $HAPLN1^{low}$ n=70, $HAPLN1^{high}$ n=70; Epithelial: $HAPLN1^{low}$ n=51, $HAPLN1^{high}$ n=48; Immune: $HAPLN1^{low}$ n=52, $HAPLN1^{high}$ n=48;

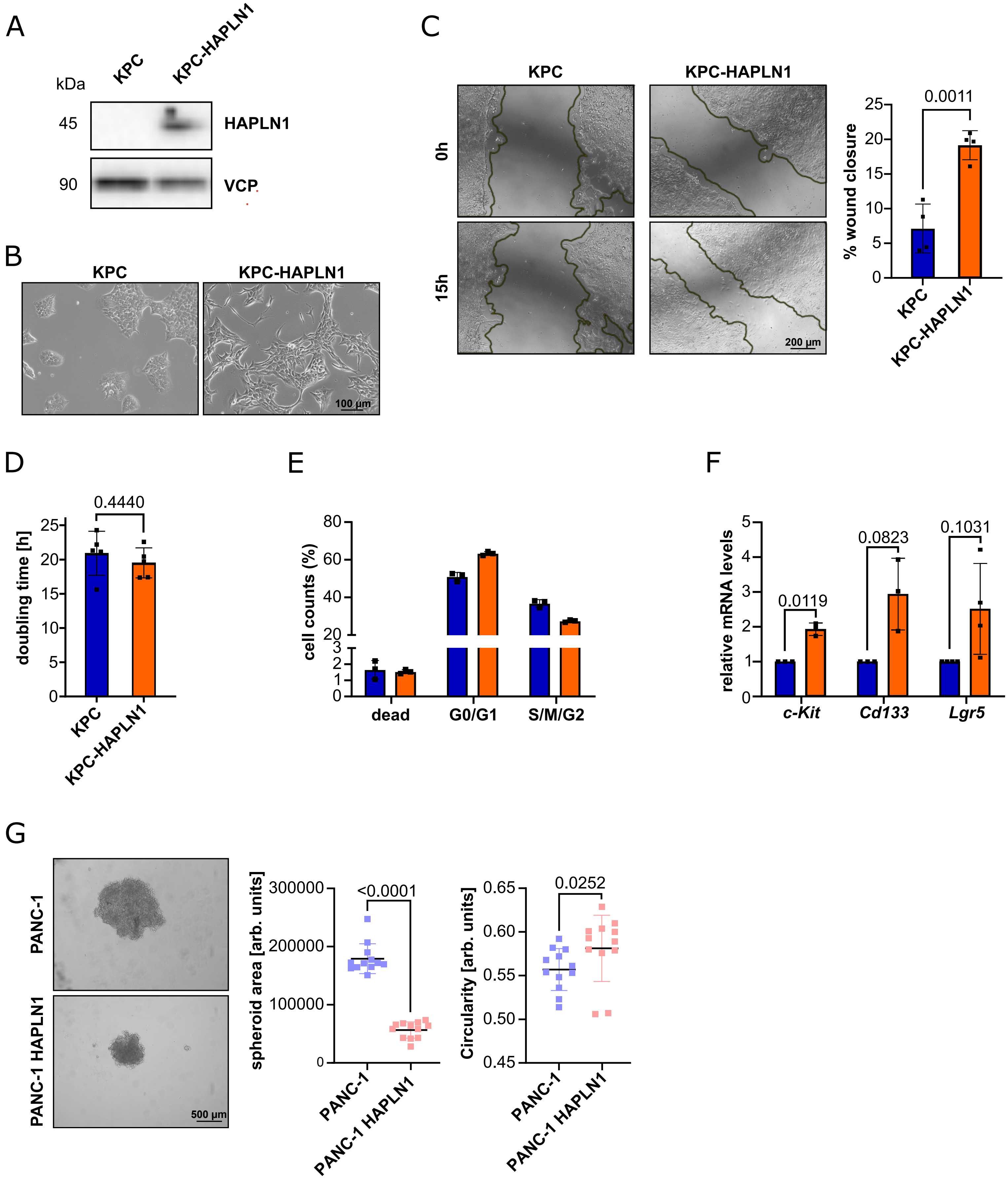

Supplementary Figure 2

Supplementary Figure 2: HAPLN1 induces EMT and ECM remodeling *in vitro*

A. Confirmation of HAPLN1 overexpression by Western blot analysis with VCP as house keeper. Representative image shown (n=2). **B**. Cellular morphology of stably transfected cells. Scale bar: 100 µm. **C**. Scratch assay of KPC and KPC-HAPLN1 cells for 15 h. Closure was monitored by live cell imaging. Borders in the beginning (0 h) and in the end (15 h) are marked. n=4. Scale bar: 200 µm. **D**. Doubling time of KPC and KPC-HAPLN1 assessed by cell counting. n=4. **E**. Cell cycle analysis of KPC (blue) and KPC-HAPLN1 (orange) cells. n=3. **F**. mRNA expression of stemness markers by qRT-PCR. n=3. **G**. PANC1 cells seeded in ultra-low attachment plates. Pictures taken after 48 h. Quantification of area as measure for proper spheroid formation. Scale bar 500 µm. n=12. Graphs display mean±SD. Data points mark independent biological replicates. For all data, unpaired two-tailed T test was applied, except for F where we performed paired two-tailed T test.

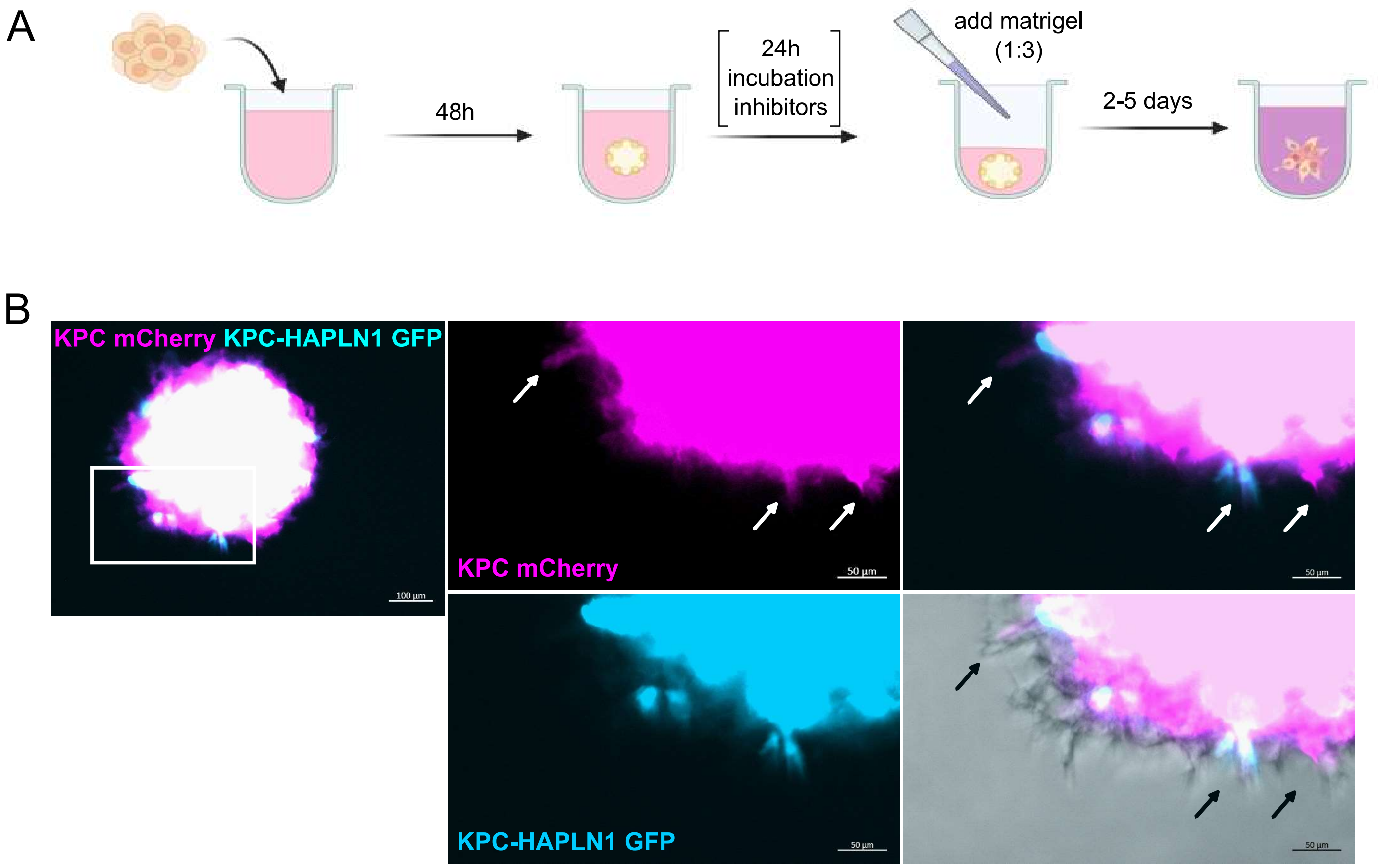

Supplementary Figure 3

Supplementary Figure 3: HAPLN1 expression affects also neighboring cells

A. Schematic overview of spheroid formation, possible treatment and embedding into Matrigel to assess invasive capacity. **B**. KPC and KPC-HAPLN1 cells were labeled by adenoviral infection with mCherry or GFP. Invasion was assessed after 48 h. Representative images of 3 independent experiments are shown. Scale bar: 100 µm, zoom: 50 µm.

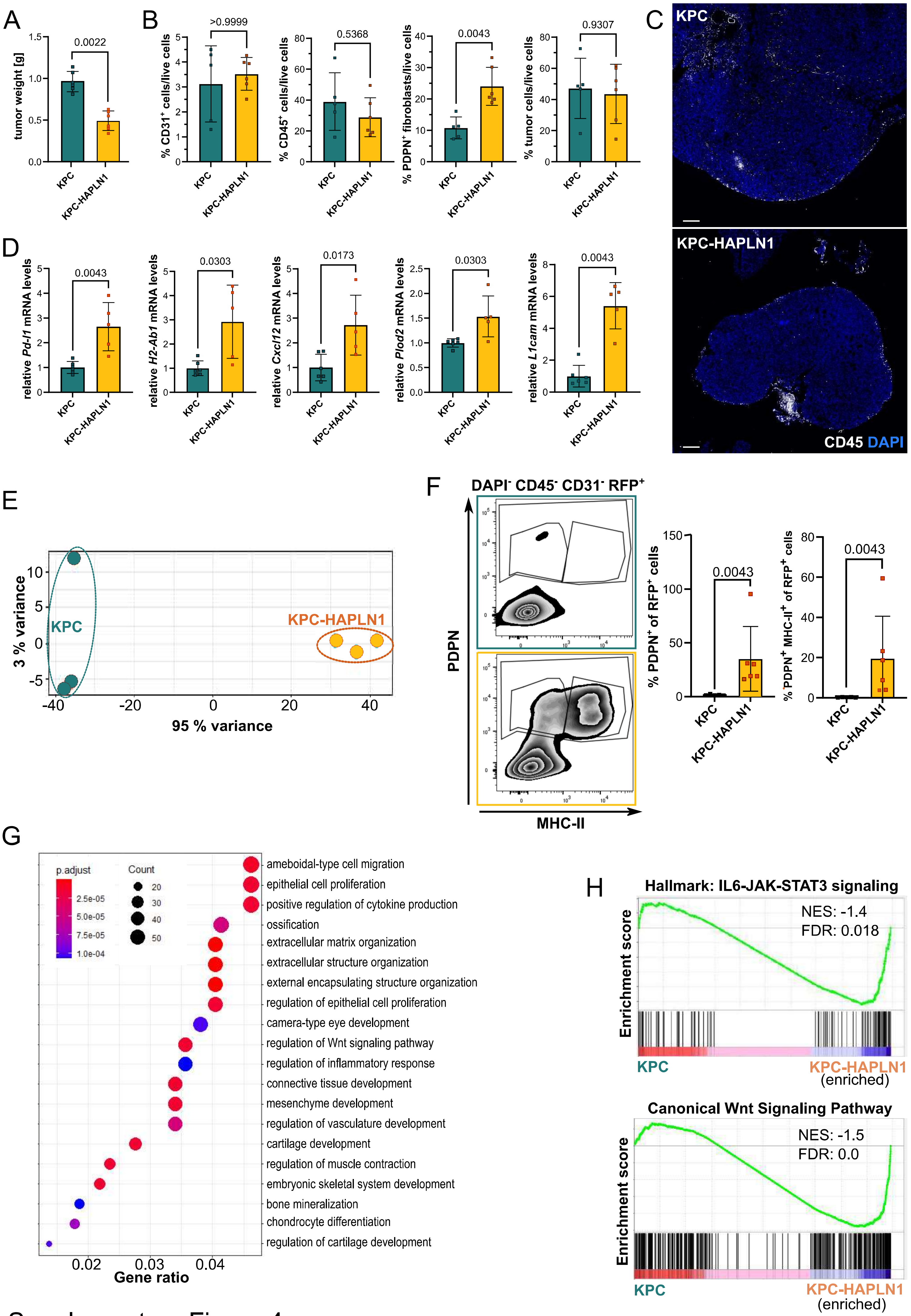

Supplementary Figure 4

Supplementary Figure 4: HAPLN1 induces high plastic tumor cells *in vivo*

A. Solid tumor weight after 11 days of tumor growth. n=6. **B**. Analysis of tumor composition after digestion by flow cytometry. KPC n=5; KPC-HAPLN1 n=6. **C**. Immunofluorescence staining for CD45. One tumor nodule shown as representative image. KPC n=5; KPC-HAPLN1 n=6. **D**. qRT-PCR analysis of whole tumor mRNA. KPC n=6; KPC-HAPLN1 n=5. **E**. Principal component analysis (PCA) of isolated tumor cells send for RNA sequencing. Replicates of the same group are marked with same color. PC=principal component. **F**. Flow cytometric analysis of digested tumor samples. Representative plots of MHC-II and PDPN expression by tumor cells and quantification. KPC n=5; KPC-HAPLN1 n=6. **G.** Gene Ontology for 'Biological Process' of genes significantly upregulated in KPC-HAPLN1 cells compared to KPC. n=3. **H**. GSEA of RNAseq data of isolated tumor cells on "Hallmark of IL6-JAK-STAT3 signaling" and "Canonical Wnt Signaling Pathway" gene sets. Data points represent independent biological replicates, data shown is mean±SD. Non-parametric two-tailed Mann-Whitney U test was applied.

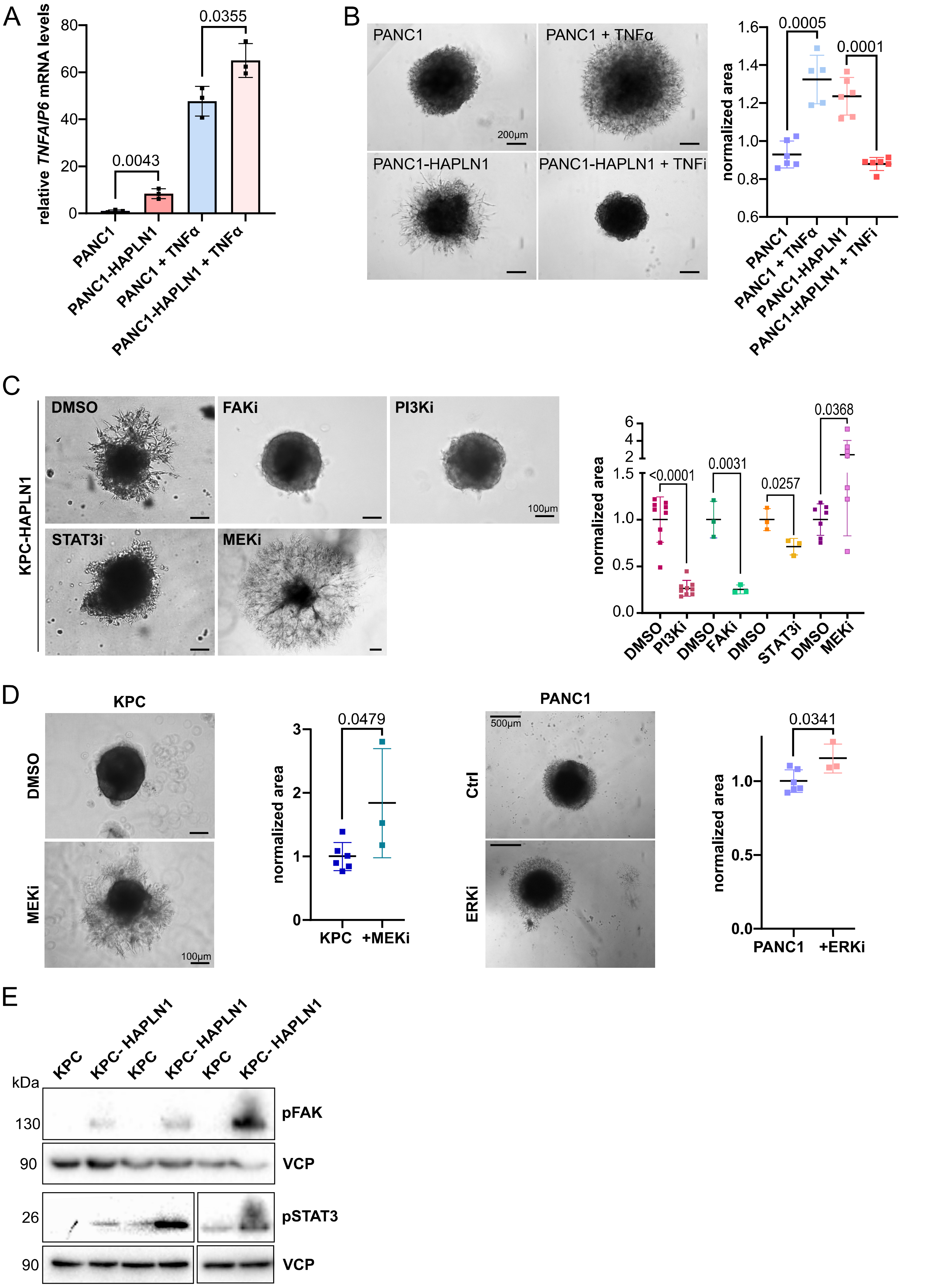

Supplementary Figure 5

Supplementary Figure 5: Increased TNF signaling induces tumor cell plasticity

A. Analysis of TNF-induced gene expression *in vitro*. Stimulation with 50 ng/ml recombinant TNFα for 6 h in starvation. n=4. **B**. Invasion assay into Matrigel of PANC1 stimulated or not with 50 ng/ml TNFα (upper panel) and PANC1-HAPLN1 treated or not with TNFα antagonist (lower panel). Invasion was measured as normalized occupied area 72 h after embedding. n=5. Scale bar: 500 µm. **C**. KPC-HAPLN1 spheroids were pretreated with inhibitors before embedding into Matrigel. Invasion was assessed 72-96 h later. PI3Ki=10 µM LY294002; FAKi=10 µM GSK2256098; STAT3i=25 µM Stattic; MEKi=50 µM PD98059. Representative images (scale bar: 100 µm) and quantification by assessing occupied area (PI3Ki n=9; FAKi, STAT3i n=3; ERKi n=7) are shown. **D**. KPC (left; n=6) or PANC1 (right; n=6) spheroids embedded in Matrigel after 48 h. Spheroids were pre-treated with DMSO, MEK inhibitor (MEKi; 50 µM PD98059) (KPC; n=3) or ERK inhibitor (ERK Activation Inhibitor Peptide I; 1 µM) (PANC1; n=3). Representative image shown. Scale bar: 100 µm (KPC), 500 µm (PANC1). **E.** Western blot analysis of STAT3 and FAK phosphorylation of untreated KPC or KPC-HAPLN1 cells. n=3. Graphs represent mean±SD. Data points indicate independent biological replicates. Unpaired two-tailed T test was used for analysis of panels A-D.

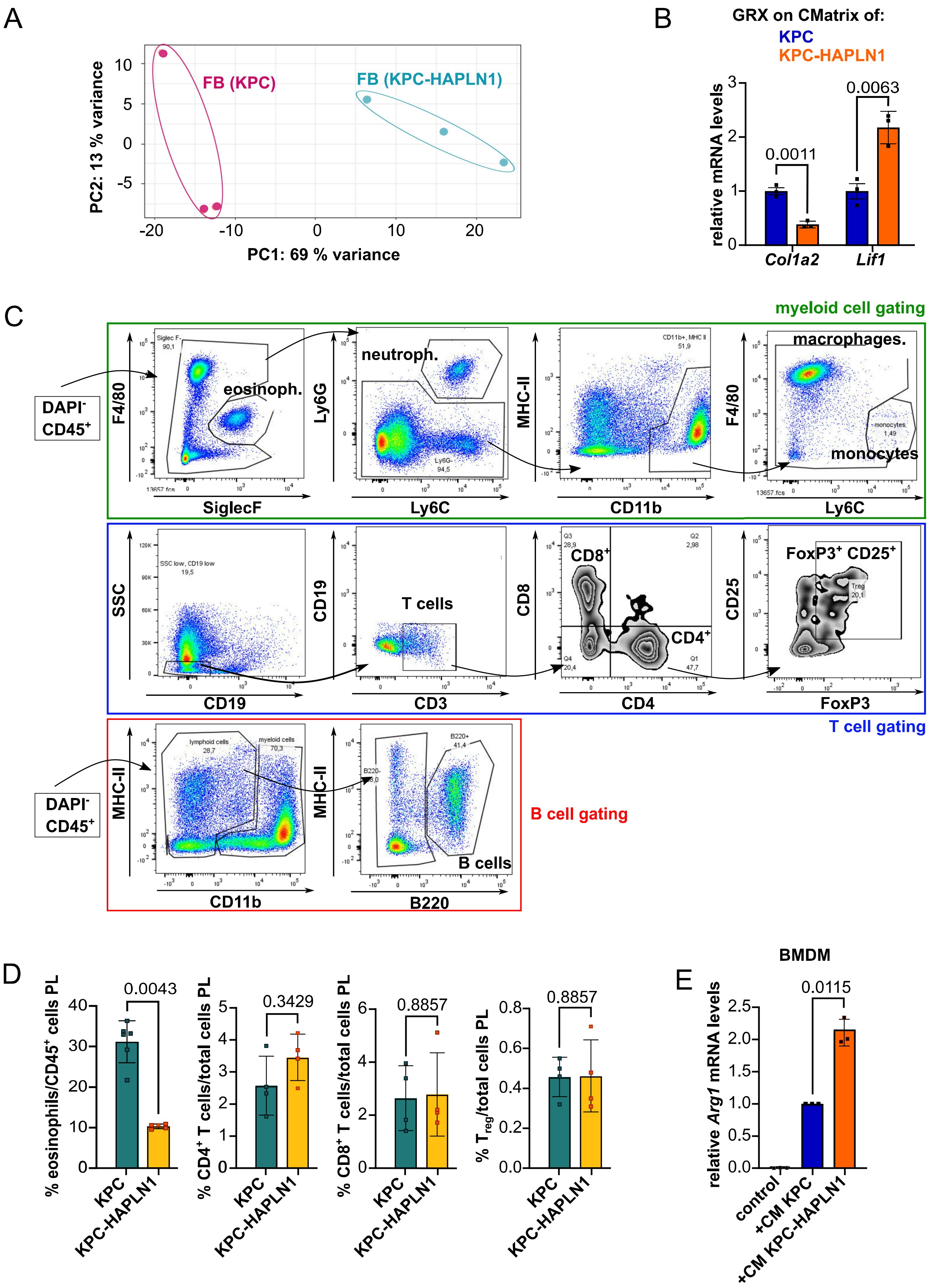

Supplementary Figure 6

Supplementary Figure 6: HAPLN1 modifies the immune microenvironment. A. Principal component analysis (PCA) of isolated fibroblasts (FB) of KPC or KPC-HAPLN1 tumors. **B**. GRX fibroblasts were cultured for 24 h on CMatrix of KPC or KPC-HAPLN1 cells. Gene expression of *Lif* and *Col1a2* are depicted. n=3. **C**. Gating of flow cytometric analysis of immune cell populations. Representative sample shown. **D.** Immune cell populations in the peritoneum assessed by flow cytometry. KPC n=5; KPC-HAPLN1 n=6, for eosinophils; n=4 for T cells. **E.** Bone marrow-derived macrophages (BMDMs) were treated with conditioned medium (CM) of KPC or KPC-HAPLN1 tumor cells for 24 h. Gene expression of *Arg1* was analyzed by qRT-PCR. n=3. Data points represent independent biological replicates, data shown is mean±SD. Non-parametric two-tailed Mann-Whitney U test was applied for panel D, unpaired two-tailed T test for panel B and paired two-tailed T test for panel E.

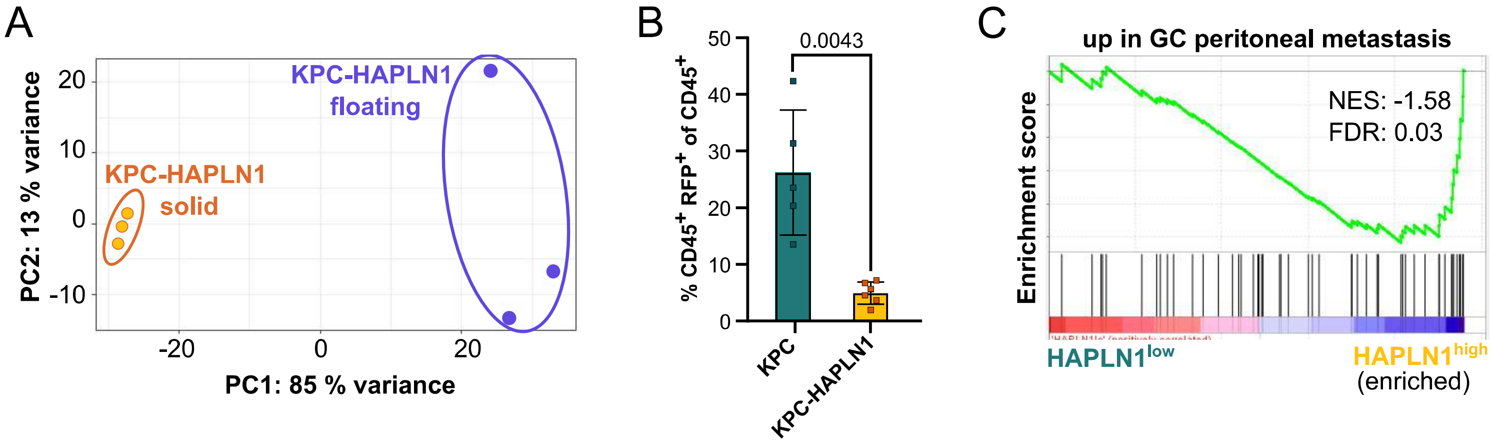

Supplementary Figure 7

Supplementary Figure 7: HAPLN1 facilitates peritoneal colonization by tumor cells

A. KPC-HAPLN1 cells from solid tumor and in suspension were isolated and send for RNAseq. Principal Component analysis (PCA) of the samples. n=3. **B**. Flow cytometric analysis of percentage of CD45+ RFP+ cells of immune cells after doublet exclusion. KPC n=5; KPC-HAPLN1 n=6. **C**. GSEA of GSE62452, dividing patients into HAPLN1 high/low according to their mean HAPLN1 expression. A gene set for genes upregulated in gastric cancer peritoneal metastasis was analyzed[39]. n=69. Data points represent independent biological replicates, data shown is mean±SD. Non-parametric two-tailed Mann-Whitney U test was applied for panel B.

Supplementary Movie 1. Scratch assay of KPC cells. KPC cells were cultured until confluence was achieved. Movie shows the migration on the scratch during 16 h.

Supplementary Movie 2. Scratch assay of KPC-HAPLN1 cells. KPC-HAPLN1 cells were cultured until confluence was achieved. Movie shows the migration on the scratch during 16 h.
