# Supplementary material for: HAPLN1 potentiates peritoneal metastasis in pancreatic cancer
Source: Nat Commun. 2023 Apr 24;14:2353. doi: 10.1038/s41467-023-38064-w (PMC10126109; doi:10.1038/s41467-023-38064-w)
Supplement: Supplementary file 3 — Description of Additional Supplementary Files [file 41467_2023_38064_MOESM3_ESM.pdf]

Description of Additional Supplementary Files

File Name: Supplementary Movie 1
Description: **Scratch assay of KPC cells.** KPC cells were cultured until confluence was achieved. Movie shows the migration on the scratch during 16 h.

File Name: Supplementary Movie 2
Description: **Scratch assay of KPC-HAPLN1 cells.** KPC-HAPLN1 cells were cultured until confluence was achieved. Movie shows the migration on the scratch during 16 h.
