# Supplementary material for: HAPLN1 potentiates peritoneal metastasis in pancreatic cancer
Source: Nat Commun. 2023 Apr 24;14:2353. doi: 10.1038/s41467-023-38064-w (PMC10126109; doi:10.1038/s41467-023-38064-w)
Supplement: Supplementary file 6 — Reporting Summary [file 41467_2023_38064_MOESM6_ESM.pdf]

nature portfolio

Corresponding author(s): Andreas Fischer; Juan Rodriguez-Vita

Last updated by author(s): Aug 9, 2022

Reporting Summary

Nature Portfolio wishes to improve the reproducibility of the work that we publish. This form provides structure for consistency and transparency in reporting. For further information on Nature Portfolio policies, see our Editorial Policies and the Editorial Policy Checklist.

Statistics

For all statistical analyses, confirm that the following items are present in the figure legend, table legend, main text, or Methods section.

n/a	Confirmed	
☐	☒	The exact sample size (*n*) for each experimental group/condition, given as a discrete number and unit of measurement
☐	☒	A statement on whether measurements were taken from distinct samples or whether the same sample was measured repeatedly
☐	☒	The statistical test(s) used AND whether they are one- or two-sided<br>*Only common tests should be described solely by name; describe more complex techniques in the Methods section.*
☒	☐	A description of all covariates tested
☐	☒	A description of any assumptions or corrections, such as tests of normality and adjustment for multiple comparisons
☐	☒	A full description of the statistical parameters including central tendency (e.g. means) or other basic estimates (e.g. regression coefficient) AND variation (e.g. standard deviation) or associated estimates of uncertainty (e.g. confidence intervals)
☐	☒	For null hypothesis testing, the test statistic (e.g. *F*, *t*, *r*) with confidence intervals, effect sizes, degrees of freedom and *P* value noted<br>*Give P values as exact values whenever suitable.*
☒	☐	For Bayesian analysis, information on the choice of priors and Markov chain Monte Carlo settings
☒	☐	For hierarchical and complex designs, identification of the appropriate level for tests and full reporting of outcomes
☒	☐	Estimates of effect sizes (e.g. Cohen's *d*, Pearson's *r*), indicating how they were calculated

Our web collection on statistics for biologists contains articles on many of the points above.

Software and code

Policy information about availability of computer code

Data collection	Data was colletected with NextSeq 550 PE 75 HO (Illumina), BD FACSDiva, Quantstudio, Clariostar.
Data analysis	RNA-sequencing data sets was performed using the software R using the DESeq2 package. Plots were created using ggplot2 or EnhancedVolcano. apeglm package was used for LFC shrinkage. Statistical analysis was performed using the GraphPad Prism 9 software.

For manuscripts utilizing custom algorithms or software that are central to the research but not yet described in published literature, software must be made available to editors and reviewers. We strongly encourage code deposition in a community repository (e.g. GitHub). See the Nature Portfolio guidelines for submitting code & software for further information.

Data

Policy information about availability of data

All manuscripts must include a data availability statement. This statement should provide the following information, where applicable:

- Accession codes, unique identifiers, or web links for publicly available datasets
- A description of any restrictions on data availability
- For clinical datasets or third party data, please ensure that the statement adheres to our policy

RNAseq data have been uploaded to the Gene Expression Omnibus (GEO) database.

Human research participants

Policy information about studies involving human research participants and Sex and Gender in Research.

Reporting on sex and gender	All patient data in this manuscript come from publicly available databases.
Population characteristics	*Describe the covariate-relevant population characteristics of the human research participants (e.g. age, genotypic information, past and current diagnosis and treatment categories). If you filled out the behavioural & social sciences study design questions and have nothing to add here, write "See above."*
Recruitment	*Describe how participants were recruited. Outline any potential self-selection bias or other biases that may be present and how these are likely to impact results.*
Ethics oversight	*Identify the organization(s) that approved the study protocol.*

Note that full information on the approval of the study protocol must also be provided in the manuscript.

Field-specific reporting

Please select the one below that is the best fit for your research. If you are not sure, read the appropriate sections before making your selection.

☒ Life sciences ☐ Behavioural & social sciences ☐ Ecological, evolutionary & environmental sciences

For a reference copy of the document with all sections, see nature.com/documents/nr-reporting-summary-flat.pdf

Life sciences study design

All studies must disclose on these points even when the disclosure is negative.

Sample size	Details regarding the sample size of all experiments are provided in Methods section and figure legends. Reasonable sample sizes were chosen to ensure they are sufficient for statistical comparison between different groups.
Data exclusions	No data were excluded from the analyses.
Replication	In vitro experiments were completed at least in triplicate to successfully verify reproducibility.
Randomization	All groups were asigned randomly. Littermate controls were used to make covariates not relevant.
Blinding	Blind data collection and analysis was implemented.

Reporting for specific materials, systems and methods

We require information from authors about some types of materials, experimental systems and methods used in many studies. Here, indicate whether each material, system or method listed is relevant to your study. If you are not sure if a list item applies to your research, read the appropriate section before selecting a response.

Materials & experimental systems

n/a	Involved in the study	
☐	☒	Antibodies
☐	☒	Eukaryotic cell lines
☒	☐	Palaeontology and archaeology
☐	☒	Animals and other organisms
☒	☐	Clinical data
☒	☐	Dual use research of concern

Methods

n/a	Involved in the study	
☒	☐	ChIP-seq
☐	☒	Flow cytometry
☒	☐	MRI-based neuroimaging

Antibodies

Antibodies used	For western blot:<br>anti-HAS2 (1:500, Abcam, ab140671)<br>anti-VCP (1:5000, Abcam, ab109240)<br>anti-ACTB (1:5000, Sigma Aldrich, A5441)

anti-LRRC15 (1:1000, Abcam, ab150376)
anti-E-Cadherin (1:2000, BD Biosciences, 610181)
anti-HAPLN1 (1:1000, Bio-Techne, AF2608)
Secondary antibodies goat anti-rabbit HRP
rabbit anti-mouse HRP (1:2500, DAKO, P0448, P0260)
donkey anti-goat HRP (1:2500, R&D, HAF109)
For flow cytometry:
B220-APC 1:100 BioLegend 103211
CD11b-BUV805 1:800 BD Biosciences 741934
CD11b-PE-Cy7 1:800 BD Biosciences 552850
CD19-Alexa700 1:50 BD Biosciences 557958
CD25-PE 1:200 Life Technologies 12-0251-82
CD31-APC 1:100 BD Biosciences 561814
CD3-PerCp-Cy5.5 1:50 BioLegend 100217
CD45-PerCp-Cy5.5 1:200 BD Biosciences 550994
CD45-FITC 1:400 BD Biosciences 553079
CD4-PE-Cy7 1:300 Life Technologies 25-0041-82
CD8-APC-Cy7 1:100 BD Biosciences 557654
DAPI (stock: 10,5 mM) 1:10.000 Life Technologies D3571
F4/80-Alexa Fluor 488 1:100 Life Technologies 53-4801-82
FoxP3-Alexa Fluor 488 1:50 BD Biosciences 560403
Ly6C-PE 1:200 BD Biosciences 560592
Ly6G-APC-Cy7 1:100 BD Biosciences 560600
MHC-II-BV510 1:100 BD Biosciences 742893
PDPN-APC-Cy7 1:100 BioLegend 127417
SiglecF-BUV395 1:100 BD Biosciences 740280

Validation	All antibodies were validated by the manufacturers for antigen specificity and species reactivity as shown in the data sheets and attached references for each catalogue number.

Eukaryotic cell lines

Policy information about cell lines and Sex and Gender in Research

Cell line source(s)	KPC cell line was provided by Prof. Stephen Konieczny, at Purdue University
Authentication	The cell lines were morphologically confirmed according to the information provided by Prof. Konieczny.
Mycoplasma contamination	All cell lines showed negative for mycoplasma contamination
Commonly misidentified lines (See ICLAC register)	No commonly misidentified cell lines were used in this study

Animals and other research organisms

Policy information about studies involving animals; ARRIVE guidelines recommended for reporting animal research, and Sex and Gender in Research

Laboratory animals	For mouse experiments, 9-11 weeks female C57BL/6J mice were purchased from Janvier Labs.
Wild animals	No wild animals were used in this study
Reporting on sex	Sex was not consider to have a role on the effects observed.
Field-collected samples	This study did not involve field-collected samples.
Ethics oversight	All mouse experiments were approved by the local authorities (RP Karlsruhe and DKFZ) and carried out following their legal requirements and in accordance to the 3Rs principles.

Note that full information on the approval of the study protocol must also be provided in the manuscript.

Flow Cytometry

Plots

Confirm that:

☐ The axis labels state the marker and fluorochrome used (e.g. CD4-FITC).

☒ The axis scales are clearly visible. Include numbers along axes only for bottom left plot of group (a 'group' is an analysis of identical markers).

☒ All plots are contour plots with outliers or pseudocolor plots.

☒ A numerical value for number of cells or percentage (with statistics) is provided.

Methodology

Sample preparation	For flow cytometry and cell sorting, solid tumors were digested using 2,5 mg/ml Collagenase D (Sigma Aldrich, 11088866001), 0,5 mg/ml Liberase DL (Sigma Aldrich, 5466202001) and 0,2 mg/ml DNase (Sigma Aldrich, 10104159001) in 10 % FCS DMEM for 45 min at 37 °C after mincing thoroughly. If peritoneal lavage contained erythrocytes, red blood cell lysis was performed with RBC Lysis Buffer (Life Technologies, 00-4333-57).
Instrument	Analysis by flow cytometry was performed on a BD LSR Fortessa, while BD FACS Aria/BD FACS Aria Fusion Sorters were used for cell sorting.
Software	FlowJo Software was used for analysis of the acquired samples.
Cell population abundance	Solid tumors contained a mean of 40% tumor cells, determined as CD45-/CD31-/RFP+. Fibroblasts content was between 10 and 20% of live cells, determined by CD45-/CD31-/RFP-/PDPN+. Macrophages were 15% or 45% of the immune populations in the peritoneal cavity, determined by CD45+/CD11b-high/F4/80+. Stringent gating strategies were applied to ensure purity.
Gating strategy	Tumor cells: DAPI-/CD45-/CD31-/RFP+.<br>Fibroblasts: DAPI-/CD45-/CD31-/RFP-/PDPN+<br>Macrophages: DAPI-/CD45+/CD11b-high/F4/80+<br>Neutrophils: DAPI-/CD45+/CD11b+/Ly6G+<br>Monocytes: DAPI-/CD45+/CD11b+/Ly6Chigh<br>B cells: DAPI-/CD45+/CD11b- and int/MHC-II+/B220+<br>Eosinophils: DAPI-/CD45+/CD11b+/SiglecF+

☒ Tick this box to confirm that a figure exemplifying the gating strategy is provided in the Supplementary Information.
